# Association between use of vitamin and mineral supplement and non-alcoholic fatty liver disease in hypertensive adults

**DOI:** 10.1038/s41598-023-40868-1

**Published:** 2023-08-22

**Authors:** Yoonmi Park, Stephanie A. Smith-Warner, Xuehong Zhang, Yoon Jung Park, Hyesook Kim, Hyesook Park, Hye Ah Lee, Seungyoun Jung

**Affiliations:** 1 Department of Nutritional Science and Food Management, Ewha Womans University, Seoul, 03760 Republic of Korea; 2 Graduate Program in System Health Science and Engineering, Ewha Womans University, Seoul, Republic of Korea; 3 Department of Nutrition, Harvard T. H. Chan School of Public Health, Boston, MA USA; 4 Department of Epidemiology, Harvard T. H. Chan School of Public Health, Boston, MA USA; 5 Channing Division of Network Medicine, Department of Medicine, Brigham and Women’s Hospital and Harvard Medical School, Boston, MA USA; 6 Department of Food and Nutrition, Wonkwang University, Jeonbuk, Republic of Korea; 7 Department of Preventive Medicine, College of Medicine, Ewha Womans University, Seoul, Republic of Korea; 8 Clinical Trial Center, Mokdong Hospital, Ewha Womans University, Seoul, Republic of Korea

**Keywords:** Diseases, Medical research, Risk factors

## Abstract

Non-alcoholic fatty liver disease (NAFLD) is the most common hepatic metabolic disorder in hypertensive adults. Impaired metabolism of micronutrients may increase NAFLD risk by exacerbating oxidative stress, insulin resistance, and inflammation among hypertensive adults. In this first cross-sectional analysis of 7,376 hypertensive adults with 2,015 NAFLD cases in the Korea National Health and Nutrition Examination Survey, vitamin and mineral supplements (VMS) use was identified via questionnaire. NAFLD was defined by a hepatic steatosis index > 36. Multivariable-adjusted odds ratios (MVOR) and 95% confidence intervals (CIs) were calculated using logistic regression models. In our study, 18.6% were current users of VMS; of these, 76.7% used multi-vitamin/mineral supplements. Current VMS users had significantly lower odds of NAFLD, compared with non-users (MVOR [95% CI]: 0.73 [0.58–0.92]). The inverse association became attenuated and non-significant among those consuming VMS at higher frequency (≥ 2 times/day), for longer duration (> 16 months), and taking ≥ 2 VMS products. The inverse association with current use of VMS was only evident in those aged < 56 years (MVOR [95% CI]: 0.54 [0.40–0.72]) and men (MVOR [95% CI]: 0.56 [0.40–0.80])(*P*_interaction_ ≤ 0.04). Our results suggest that VMS use may lower NAFLD risk, particularly among younger or male hypertensive adults, if taken in moderation.

## Introduction

Approximately 1.28 billion people, accounting for 30% of the adult population worldwide, were estimated to have hypertension, also known as elevated blood pressure, in 2019^1^. Importantly, 49.5% of hypertensive adults are reported to suffer from the non-alcoholic fatty liver disease (NAFLD)^2,3^, the excessive accumulation of fat or fibrosis progression in the liver, that leads to deadly cirrhosis, hepatocellular carcinoma, and liver and cardiovascular death^4^. The reason for this comorbidity remains unclear, but hypertension-induced inflammation or abnormal activation of the renin-angiotensin system are hypothesized to predispose individuals to insulin resistance^2,3,5,6^, a strong risk factor for NAFLD.

Vitamin and mineral supplements (VMS) are the most commonly used dietary supplement worldwide. According to a national representative study in South Korea, 34.2% of adults were found to use dietary supplements; of these, 75% were VMS users^7^. Similar use of VMS has been reported in Western countries^8,9^. Moreover, use of VMS or other dietary supplements has been observed to be greater among individuals with chronic disease conditions, including hypertension^10^.

Of note, impaired metabolism of micronutrients is thought to increase susceptibility to NAFLD, possibly by exacerbating the lipotoxic hepatic environment, oxidative stress, insulin resistance, necro-apoptosis, and the immune system^11^, supporting the use of VMS to prevent NAFLD. Indeed, a majority of previous serum or plasma studies of single vitamins and minerals (e.g., vitamins A^12,13^, C^13,14^, D^13,15–18^, E^12,13^, folate^19,20^, selenium^21,22^, ferritin^23,24^) or multivitamins^13^ have reported significant inverse associations, though not all, with the risk of NAFLD^13–24^ or NAFLD severity^15,21,23^. However, prior studies on supplement use have focused on NAFLD prognosis among NAFLD patients^25,26^. No study has yet examined the association between VMS use and NAFLD among hypertensive individuals, a group who are at greater risk of hypertension-induced metabolic abnormalities associated with NAFLD and, thus, might have varied clinical benefit from VMS assisting in metabolic regulation^2,3,5,6^ to the general population as well as NAFLD patients.

Therefore, we evaluated the potential benefit or harm of VMS associated with NAFLD risk among hypertensive adults. Specifically, we explored the appropriate use of VMS by integrating the large resources of VMS uses, lifestyle factors, and medical conditions in the Korea National Health and Nutrition Examination Survey (KNHANES)^27^. Comprehensive analyses were conducted to examine associations across diverse domains of VMS use, including status, frequency, duration, and products number in use, and among subgroups defined by demographics, lifestyle, and clinical factors.

## Methods

### Study design and study population

We analyzed data from the KNHANES 2005 and 2007–2009. The KNHANES is an annual national cross-sectional survey conducted by the Korea Centers for Disease Control and Prevention (KCDC) to assess the health and nutritional status of Korean^27^. Lifestyle and health information was obtained from a health behavior survey, a health examination survey, and a nutrition survey. Trained staff conducted standardized physical examinations and laboratory tests in a mobile examination center. Informed consent was obtained from all participants prior to their enrollment in the KNHANES. The study was conducted according to the guidelines of the Declaration of Helsinki. All KNHANES protocols were approved by the KCDC Research Ethics Review Committee and the study protocol for the present analyses was approved by the Institutional Review Board of Ewha Womans University (IRB no. ewha-202209–0011-01).

**Figure 1:**
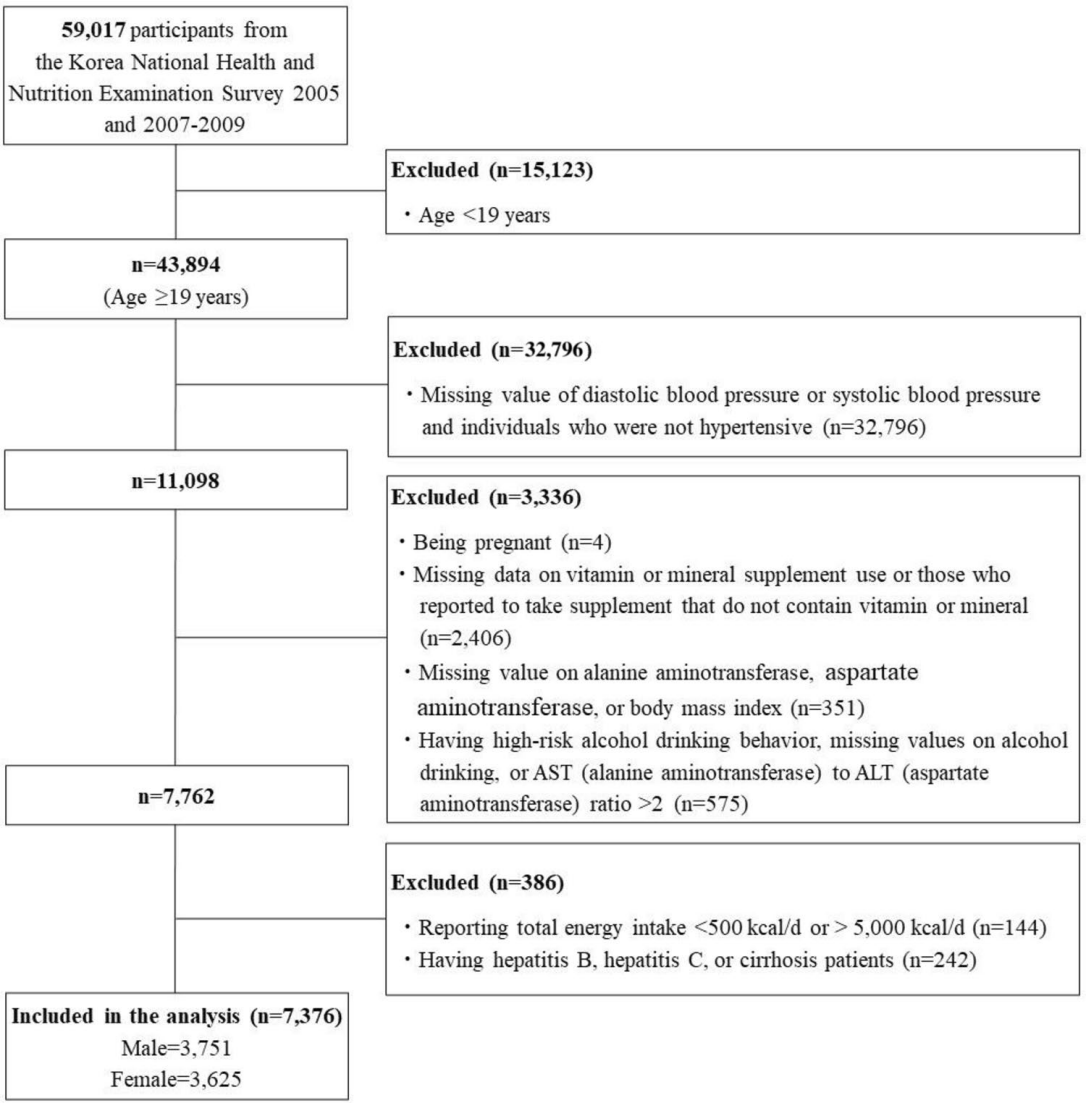
Flow diagram of subject inclusion and exclusion.

The study population was restricted to hypertensive adults. Hypertension was defined as (1) having an average value from three measurements of systolic blood pressure ≥ 130 mmHg or of diastolic blood pressure ≥ 80 mmHg, or (2) using hypertension medications^28^. Of 59,017 individuals who participated in the KNHANES 2005 and 2007–2009, we identified 11,098 hypertensive adults aged ≥ 19 years. Participants who met the following conditions were excluded from the analyses: (1) being pregnant (n = 4), (2) missing data on VMS use or reporting diet supplement products not containing vitamins or minerals (n = 2,406), (3) having no serum alanine aminotransferase (ALT), serum aspartate aminotransferase (AST), or body mass index (BMI) data which were used to ascertain NAFLD (n = 351), (4) having an AST to ALT ratio > 2 suggestive of alcoholic liver disease^29^, having high-risk alcohol drinking behaviors defined by consuming alcoholic beverages > 26 cups/week for men and 17.5 cups/week for women^30^, or missing data on alcohol consumption due to the possibility of alcoholic fatty liver disease (n = 575), (5) consuming implausible energy intake (< 500 kcal/d or > 5,000 kcal/d) (n = 144), or (6) having hepatitis B, hepatic C, or hepatocirrhosis (n = 242). Consequently, our analyses included 7376 adults (Fig. 1).

### Assessment of VMS use

VMS use was asked as a part of a nutritional survey conducted by staff visiting participants’ homes^27^. The questionnaire on diet supplement use inquired about VMS use during the past year with sub-questions on frequency (≥ 3 times/day, 2 times/day, 1 time a day, 2–5 times/week, ≤ 1 time/week, and not at all) and duration (open-ended question) of use for up to four different VMS. The VMS products’ names and manufacturers were also asked via face-to-face interviews. We confirmed the nutrient composition of the VMS by searching for product names in the Drug Info (www.Druginfo.co.kr), Ministry of Food and Drug Safety (foodsafetykorea.go.kr), and Korea Pharmaceutical Information Center (www.health.kr) websites or those of the respective manufacturing companies.

For the study, we defined current nonusers of VMS as those who answered “no” to the question, ‘in the past year, have you consumed any supplements for at least 2 weeks on a continuous basis?’. Current users of VMS were defined as those who met the following criteria: (1) answered “yes” to the question on current VMS use and (2) reported VMS products that were confirmed to contain vitamins and minerals through our nutrient composition searches of the products. We estimated the frequency and duration of VMS use by taking the maximal frequency and duration of all VMS reported. In addition, using the reported number of VMS products, we classified VMS users into users of one VMS and users of two or more VMS. Finally, we grouped VMS users into three groups reflecting the micronutrient composition of the VMS used, namely multivitamin or multimineral supplement (M_VMS), single-vitamin supplement (S_VIT), and single-mineral supplement (S_MIN) users.

### Other data collection

Information on sociodemographic (e.g., age, sex, education, and household income) and health-related lifestyle (smoking habits, alcohol drinking, physical activity) factors were collected through self-reported questionnaires^27^. Regular exercise was further defined as being engaged in ≥ 5 days/week of at least 30 min moderate-intensity activities and/or ≥ 3 days/week of at least 20 min vigorous-intensity activities^31^. Dietary intake was assessed via a 24-h dietary recall and converted into nutrient intake using the national standard food composition table^28^. Body weight, height, and waist circumference were measured. Blood testing was performed using serum samples taken after at least 8 h overnight fasting that had been immediately refrigerated and shipped to the Central Testing Institute within 24 h. High density lipoprotein cholesterol (HDL-C), triglyceride, and glucose concentrations were measured using a Hitachi 700–110 chemistry analyzer (Hitachi, Tokyo, Japan). The ALT and AST were measured using an ADIVIA 1650 analyzer (Siemens, Washington, DC, USA) up to 2007 and a Hitachi automatic analyzer 7600 (Hitachi, Tokyo, Japan) thereafter. The presence of diabetes was ascertained if participants had serum glucose ≥ 126 mg/dL, used oral hypoglycemia agents or insulin injections, or had a physician’s diagnosis^28^. Hypercholesterolemia was defined as total serum cholesterol ≥ 240 mg/mL or currently taking lipid-lowering agents^28^. The presence of comorbidities was assessed by counting the presence of physician diagnosed major chronic diseases including diabetes, cardiovascular disease, chronic kidney disease, cancer, and chronic obstructive pulmonary disease.

### NAFLD ascertainment

NAFLD was defined using the hepatic steatosis index (HSI), one of the most-widely used, validated, noninvasive method to diagnose NAFLD, developed in Korean adults^32^. The constituents of HSI include the ALT, AST, BMI, sex, and diabetes status, and the index is calculated as follows: 8 × (ALT/AST ratio) + BMI (+ 2 if female; + 2 if had diabetes)^32^. The presence of NAFLD was defined as having an HSI value > 36^32^. The diagnostic accuracy of HSI in detecting NAFLD is known to be high, particularly in Asian populations^32–35^, with an area under receiver-operating curve of 0.81 (95% CI:0.82–0.83) and > 90% sensitivity and specificity^32^. Consequently, 2,015 NAFLD cases (27.3% of the study population) were ascertained.

### Statistical analyses

All analyses applied sampling weight to account for the stratified, multistage, clustered sampling design of the KNHANES^36^ and sampling-weighted estimates were reported in all tables, except for the number of study participants. Participants’ characteristics according to their current use of VMS were summarized using the SAS SURVEYMEANS procedure for continuous variables and the SAS SURVEYFREQ procedure for categorical variables.

The associations between VMS use (including frequency, duration, and multiple product use) and NAFLD risk were estimated by calculating odds ratios and 95% confidence intervals (CIs) using the SAS SURVEYLOGISTIC procedure. The multivariable model included potential confounding factors by adding, a priori selected, well-known risk factors for NAFLD^4^: age, sex, obesity status, waist circumference, household income, education, alcohol drinking status, smoking status, regular exercise, status of diabetes, status of hypercholesterolemia, serum triglyceride concentration, a comorbidity measure, hypertension stage, current use of hypertension medication, and intakes of total energy and fruits and vegetables (see Table 2 for the categorization of confounding variables). A missing indicator for missing responses of each covariate (< 4.6%) was created, if applicable. The P-trend for frequency and duration of VMS use was performed by modelling the median of each VMS frequency and duration category as a continuous term. Stratified analyses were conducted to assess if the association between VMS use and NAFLD differed by participants’ demographic, lifestyle, and clinical characteristics. Statistical significance for potential effect modification was tested using the Wald test of the product term between VMS use and the stratification factor.

All statistical analyses were conducted using SAS statistical software (version 9.4; SAS Institute Inc., Cary, NC) and were considered to be statistically significant if *p* value < 0.05.

## Results

In our study, 18.6% hypertensive adults were current VMS users. Compared with nonusers, current VMS users were more likely to be female, earn a higher household income, have at least college degree, not currently smoke, drink less alcohol, and consume more fruits and vegetables. They were also less likely to be obese, had fewer comorbidities, and had healthier levels of biochemical marker profiles (Table 1). Among current VMS users, the major types of VMS consumed were M_VMS (76.7%), followed by S_VIT (20.3%) and S_MIN (3.0%) (Table S1).

Among hypertensive adults, current users of VMS had significantly lower odds of NAFLD compared to nonusers; the multivariable-adjusted odds ratio (MVOR) (95% CI) was 0.73 (0.58–0.92) (Table 2). In analyses with specific types of VMS use (Table S2), significant inverse associations was observed with the use of M_VMS or S_MIN, though not with S_VIT, showing MVOR ranging from 0.34 to 0.76. The inverse association among M_VMS users, who were the majority among those who used multiple VMS products, was only evident among those who used one product, but not among those who took additionally other supplements (data not shown).

**Table 1:** Population characteristics^a^ according to the current use of VMS among hypertensive adults in the Korea National Health and Nutrition Examination Survey, 2005 and 2007–2009 (N = 7376).

Characteristics	Current use of VMS	
	Nonusers	Current users
	(N = 6003)	(N = 1373)
Demographics and SES		
Age, years	48.8 ± 0.3	50.0 ± 0.6
*Sex, %*		
Male	3,147 (64.0)	604 (55.7)
Female	2,856 (36.0)	769 (44.3)
Household income level, %^*b*^		
Quartile 1	1,719 (21.1)	292 (15.1)
Quartile 2	1,555 (27.2)	298 (20.4)
Quartile 3	1,402 (25.8)	333 (25.9)
Quartile 4	1,216 (23.8)	419 (36.5)
Missing	111 (2.2)	31 (2.1)
Education level, %		
Elementary school	2,479 (28.8)	426 (21.1)
Middle school	764 (12.0)	195 (13.4)
High school	1,741 (36.6)	415 (34.9)
College or higher	991 (22.2)	333 (30.3)
Missing	28 (0.4)	4 (0.3)
Health-related lifestyles		
Current smoking status, %		
Never	3,148 (44.1)	828 (53.6)
Past	1,445 (25.2)	321 (25.2)
Current	1,409 (30.7)	224 (21.2)
Missing	1 (0.0)	0 (0.0)
Alcoholic drinking status, %		
Never	1,057 (12.5)	276 (15.0)
Past	900 (12.4)	191 (11.9)
Current, ≤ 1 time/month	1,370 (22.4)	381 (27.5)
Current, 2– ≤ 4 times/month	1,154 (23.0)	257 (22.2)
Current, ≥ 2 times/week	1,522 (29.7)	268 (23.4)
Regular exercise, %		
No	4,441 (73.9)	1,000 (73.3)
Yes	1,562 (26.1)	373 (26.7)
Dietary factors		
Total energy intake, kcal/day	1,965 ± 15	1,969 ± 29
Fruits and vegetables intake, g/day	487 ± 7	531 ± 15
Vitamin A, μgRE/day	797.10 ± 16.76	833.56 ± 26.64
Vitamin B_1_, mg/day	1.28 ± 0.01	1.29 ± 0.03
Vitamin B_1_, mg/day	1.13 ± 0.01	1.22 ± 0.02
Vitamin C, mg/day	102.36 ± 1.54	116.67 ± 4.70
Calcium, mg/day	505.60 ± 6.92	545.89 ± 14.98
Phosphorus, mg/day	1184.72 ± 9.66	1227.99 ± 18.11
Irion, mg/day	14.58 ± 0.19	15.12 ± 0.34
Potassium, mg/day	2989.55 ± 25.40	3116.77 ± 53.84
Medical factors		
BMI (kg/m^2^)^c^, %		
< 18.5 kg/m^2^	153 (2.4)	28 (2.5)
18.5– < 23 kg/m^2^	1,648 (26.1)	443 (30.9)
23– < 25 kg/m^2^	1,528 (25.3)	388 (29.2)
≤ 25 kg/m^2^	2,674 (46.2)	514 (37.3)
Waist circumference, cm	85.1 ± 0.2	83.3 ± 0.3
Diabetes Mellitus, %		
No	4,951 (84.1)	1,150 (84.9)
Yes	852 (12.0)	184 (11.2)
Missing	200 (3.9)	39 (3.9)
Hypercholesterolemia^d^, %		
No	4,938 (82.8)	1,107 (81.8)
Yes	857 (12.7)	224 (13.9)
Missing	208 (4.5)	42 (4.3)
Comorbidity status score^e^, %		
0	4,370 (76.9)	1,012 (76.0)
1	1,146 (15.7)	277 (17.7)
2	244 (3.0)	42 (2.3)
3	41 (0.5)	3 (0.1)
≥ 4	2 (0.0)	0 (0.0)
Missing	200 (3.9)	39 (3.9)
Hypertension stage^f^, %		
Stage 1	3,102 (57.0)	774 (63.5)
Stage ≥ 2	2,332 (36.4)	437 (27.4)
Other	569 (6.6)	162 (9.1)
Current use of hypertension medications, %		
No	3,983 (74.4)	874 (70.9)
Yes	2,020 (25.6)	499 (29.1)
Biochemical markers		
ALT, IU/L	27.0 ± 0.4	24.2 ± 0.5
AST, IU/L	25.0 ± 0.2	24.1 ± 0.4
HDL-C, mg/dL	45.2 ± 0.2	47.0 ± 0.4
Fasting TG, mg/dL	160.1 ± 2.1	147.8 ± 3.6
Fasting glucose, mg/dL	100.2 ± 0.5	98.4 ± 0.7

*ALT* alanine aminotransferase, *AST* aspartate aminotransferase, *BMI* body mass index, *HDL-C* high density lipoprotein cholesterol, *SES* socio economic status, *TG* triglyceride, *VMS* vitamin and mineral supplements.

^a^Values are presented as mean ± SE or N (percentages); all results presented in this table are sampling-weighted estimates except the number of study participants.

^b^Household income level was grouped based on quartiles in our study population.

^c^BMI cut-off is based on Asian obesity criteria.

^d^Hypercholesterolemia was defined as participants having fasting total serum cholesterol level ≥ 240 mg/mL or currently taking lipid-lowering agents.

^e^Comorbidity status score was defined as 0 point to 5 points by counting 1 point each for comorbid status of NAFLD such as cancer, chronic obstructive pulmonary disease, diabetes mellitus, cardiovascular disease, and chronic kidney disease.

^f^Hypertension stage 1 was defined as 130 mmHg ≤ SBP < 140 mmHg or 80 mmHg ≤ DBP < 90 mmHg; hypertension stage ≥ 2 was defined as 140 mmHg ≤ SBP or 90 mmHg ≤ DBP; others were those who had blood pressure within the normal range, but used hypertensive medications.

**Table 2:** Age-adjusted and multivariable-adjusted^a,b^ odds ratio (OR) and 95% confidence intervals (95% CIs) of NAFLD according to current use of VMS use among hypertensive adults.

	Current use of VMS	
	Nonusers	Current users
Cases/Non-cases	1,714/4,289	301/1,072
Age-adjusted OR (95% CI)	1 (Ref)	0.65 (0.54–0.77)
MV-adjusted OR (95% CI)	1 (Ref)	0.73 (0.58–0.92)

*CI* confidence intervals, *NAFLD* non-alcoholic fatty liver disease, *OR* odds ratio, *VMS* vitamin and mineral supplements.

^a^All results presented in this table are sampling-weighted estimates except the number of study participants.

^b^Multivariable model was adjusted for age (quartiles), sex (male, female), household income level (quartiles), education level (elementary school, middle school, high school, college or higher), smoking status (never, past, current, missing), alcohol drinking status (never, past, ≤ 1 time/month, 2– ≤ 4 times/month, ≥ 2 times/week), regular exercise (no, yes), body mass index (< 18.5 kg/m^2^, 18.5– < 23 kg/m^2^, 23– < 25 kg/m^2^, ≥ 25 kg/m^2^), waist circumference (cm, continuous), diabetes mellitus (no, yes), hypercholesterolemia (no, yes), number of comorbidities (0, 1, 2, 3, ≥ 4), serum triglyceride concentration (mg/dL, continuous), hypertension stage (stage1, ≥ stage2, other), current use of hypertension medications (no, yes), and intakes of total energy (quartiles) and fruits and vegetables (quartiles).

**Figure 2:**
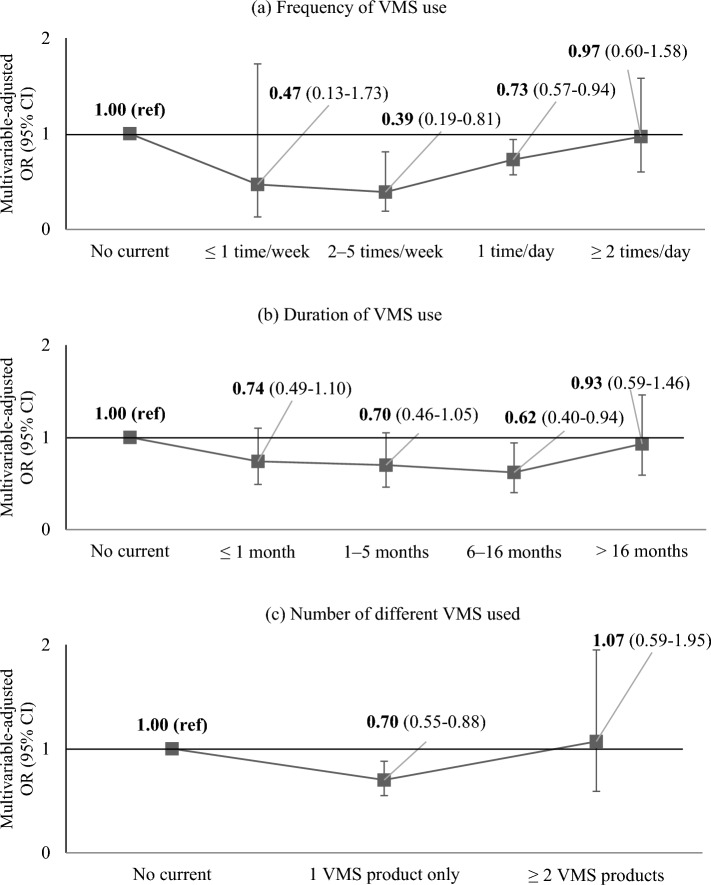
Association of NAFLD risk according to uses of vitamin and mineral supplements (VMS) defined by frequency, duration, and use of multiple products among hypertensive adults.

When we further explored associations by frequency, duration, and multiple use (Table 3, Fig. 2), we observed weak, nonsignificant association among those who took VMS at high frequency, for a long duration, or consuming multiple products. A U-shaped association was observed with frequency of VMS use where the strongest association occurred among those using VMS 2–5 times/week (MVOR [95% CI]: 0.39 [0.19–0.94]) compared with non-users. The association became null at the highest category of ≥ 2 times/day (MVOR [95% CIs]: 0.97 [0.60–1.58]). These results did not change materially when combining ≤ 1 time/week and 2– ≤ 5 times/week frequency categories or restricting the analysis to those taking only one product (data not shown). Similarly, compared with non-users, the odds of NAFLD tended to decrease with increasing duration up to 6–16 months (MVOR [95% CIs]: 0.62 [0.40–0.94]). The associations were weakened and nonsignificant for longer durations (MVOR [95% CIs]:0.93 [0.59–1.46]). There was a significant inverse association with use of a single VMS (MVOR [95% CI]: 0.70 [0.55–0.88]), but the association was not seen when multiple VMS products were taken (MVOR [95% CI]: 1.07 [0.59–1.95]), compared with non-users.

In sensitivity analyses, additional adjustment for dietary intakes of vitamins and minerals, macronutrient intakes, and eating frequency or excluding severe NAFLD cases with ≥3 BARDs score^37^ did not change our results materially (data not shown). When the comprehensive NAFLD score^34^ was used to defined NAFLD cases, the results were weakened and nonsignificant (data not shown).

There was no significant effect modification by the other factors including obesity, smoking status, alcohol consumption status, fruit and vegetable intake, stage and types of hypertension, use of hypertension medication, and blood pressure control status (Tables 4 and 5). Also, there was no significant effect modification by carbohydrate, fat, or protein intake, or eating frequency (Table S3). However, age and sex significantly modified the association between current use of VMS and odds of NAFLD (*P*_interaction_≤ 0.04) with inverse association being observed only in those aged <56 years (MVOR [95% CIs]:0.54 [0.40–0.72]) and in men (MVOR [95% CIs]: 0.56 [0.40–0.80]).

**Table 3:** Age-adjusted and multivariable-adjusted^a,b^ odds ratio (OR) and 95% confidence intervals (95% CIs) of NAFLD according to the use of VMS defined by frequency, duration, and use of multiple products among hypertensive adults.

	Frequency of VMS use					P trend
	Nonusers	≤ 1 time/week	2–5 times/week	1 time/day	≥ 2 times/day	
Cases/Non-cases	1,714/4,289	6/16	22/100	202/705	71/251	
Age-adjusted OR (95% CI)	1 (Ref)	0.81 (0.21–3.15)	0.53 (0.29–0.95)	0.61 (0.50–0.75)	0.79 (0.55–1.15)	< .001
MV-adjusted OR (95% CI)	1 (Ref)	0.47 (0.13–1.73)	0.39 (0.19–0.81)	0.73 (0.57–0.94)	0.97 (0.60–1.58)	0.13
	Duration of VMS use^d^					P trend
	Nonusers	≤ 1 month	1–5 months	6–16 months	> 16 months	
Cases/Non-cases	1,714/4,289	68/235	93/316	59/251	81/270	
Age-adjusted OR (95% CI)	1 (Ref)	0.60 (0.42–0.86)	0.66 (0.48–0.91)	0.63 (0.44–0.90)	0.70 (0.51–0.95)	0.003
MV-adjusted OR (95% CI)	1 (Ref)	0.74 (0.49–1.10)	0.70 (0.46–1.05)	0.62 (0.40–0.94)	0.93 (0.59–1.46)	0.38
	Number of different VMS					P trend
	Nonusers	1 VMS product only		≥ 2 VMS products		
Cases/Non-cases	1,714/4,289	248/929		53/143		
Age-adjusted OR (95% CI)	1 (Ref)	0.61 (0.51–0.74)		0.94 (0.65–1.37)		< 0.001
MV-adjusted OR (95% CI)	1 (Ref)	0.70 (0.55–0.88)		1.07 (0.59–1.95)		0.03

*CI* confidence intervals, *NAFLD* non-alcoholic fatty liver disease, *OR* odds ratio, *VMS* vitamin and mineral supplements.

^a^All results presented in this table are sampling-weighted estimates except the number of study participants.

^b^Multivariable model was adjusted for age (quartiles), sex (male, female), household income level (quartiles, missing), education level (elementary school, middle school, high school, college or higher), smoking status (never, past, current), alcohol drinking status (never, past, ≤ 1 time/month, 2- ≤ 4 times/month, ≥ 2 times/week), regular exercise (no, yes), body mass index (< 18.5 kg/m^2^, 18.5- < 23 kg/m^2^, 23- < 25 kg/m^2^, ≥ 25 kg/m^2^), waist circumference (cm, continuous), diabetes mellitus (no, yes), hypercholesterolemia (no, yes), number of comorbidities (0, 1, 2, 3, ≥ 4), serum triglyceride concentration (mg/dL, continuous), hypertension stage (stage1, ≥ stage2, other), current use of hypertension medications (no, yes), and intakes of total energy (quartiles) and fruits and vegetables (quartiles).

^c^P-trend was tested from model including the median value of specific VMS usage categories as a continuous term and using the Wald test of it.

^d^Duration of VMS use was categorized according to the quartiles of duration among current users of VMS.

**Table 4:** Multivariable-adjusted^a,b^ odds ratio (OR) and 95% confidence intervals (95% CIs) of NAFLD according to use of VMS according to demographic and lifestyle factors.

Stratification factors	Cases/Non-cases	Use of VMS		P interaction
		Nonusers	Current users	
		OR (95% CI)	OR (95% CI)	
By age^*d*^				
Age < 56 years	1,197/2,463	1 (Ref)	0.54 (0.40–0.72)	< .001
Age ≥ 56 years	818/2,898	1 (Ref)	1.23 (0.91–1.65)	
By sex				
Male	965/2,786	1 (Ref)	0.56 (0.40–0.80)	0.04
Female	1,050/2,575	1 (Ref)	0.99 (0.74–1.33)	
By BMI^*e*^				
BMI < 23 kg/m^2^	36/2,236	1 (Ref)	1.11 (0.43–2.86)	0.54
BMI ≥ 23 kg/m^2^	1,979/3,125	1 (Ref)	0.71 (0.56–0.90)	
By smoking status				
Never	1,113/2,864	1 (Ref)	0.90 (0.66–1.22)	0.15
Past	412/1,354	1 (Ref)	0.75 (0.47–1.20)	
Current	490/1,143	1 (Ref)	0.44 (0.27–0.72)	
By alcohol drinking status				
Never	364/969	1 (Ref)	0.85 (0.51–1.42)	0.83
Past	304/787	1 (Ref)	1.06 (0.57–1.98)	
Current	1,347/3,605	1 (Ref)	0.67 (0.51–0.88)	
By fruit and vegetable consumption^*f*^				
< 395.8 g/day	996/2,671	1 (Ref)	0.81 (0.57–1.16	0.46
≥ 395.8 g/day	1,019/2,690	1 (Ref)	0.69 (0.51–0.92)	

*BMI* body mass index, *CI* confidence intervals, *NAFLD* non-alcoholic fatty liver disease, *OR* odds ratio, *VMS* vitamin and mineral supplements.

^a^All results presented in this table are sampling-weighted estimates except the number of study participants.

^b^Multivariable model was adjusted for age (quartiles), sex (male, female), household income level (quartiles), education level (elementary school, middle school, high school, college or higher), smoking status (never, past, current), alcohol drinking status (never, past, ≤ 1 time/month, 2– ≤ 4 times/month, ≥ 2 times/week), regular exercise (no, yes), body mass index (< 18.5 kg/m^2^, 18.5– < 23 kg/m^2^, ≥ 23- < 25 kg/m^2^, ≥ 25 kg/m^2^), waist circumference (cm, continuous), diabetes mellitus (no, yes), hypercholesterolemia (no, yes), number of comorbidities (0, 1, 2, 3, ≥ 4), serum triglyceride concentration (mg/dL, continuous), hypertension stage (stage1, ≥ stage2, other), current use of hypertension medications (no, yes), and intakes of total energy (quartiles) and fruits and vegetables (quartiles).

^c^P interaction was tested by including the product term between current use of VMS and each of stratification factors.

^d^Age groups were defined using the median value of our study population as a cut-off value.

^e^BMI of 23 kg/m^2^ is the cut-off criteria to define overweight status in the Asian population.

^f^Fruit and vegetable consumption groups were categorized using the median value of our study population as a cut-off value.

**Table 5:** Multivariable-adjusted^a,b^ odds ratio (OR) and 95% confidence intervals (95% CIs) of NAFLD according to use of VMS according to population's hypertension-related factors.

Stratification factors	Cases/Non-cases	Use of VMS		P interaction
		Nonusers	Current users	
		OR (95% CI)	OR (95% CI)	
By hypertension stage^*d*^				
Stage 1	1,007/2,869	1 (Ref)	0.65 (0.48–0.88)	0.41
Stage ≥ 2	823/1,946	1 (Ref)	0.87 (0.59–1.28)	
By type of hypertension^e^				
ISH	150/659	1 (Ref)	1.41 (0.62–3.22)	0.89
IDH	860/2,142	1 (Ref)	0.62 (0.45–0.87)	
SDH	820/2,014	1 (Ref)	0.78 (0.53–1.16)	
By use of hypertension medication				
Yes	740/1,779	1 (Ref)	0.85 (0.60–1.22)	0.32
No	1,275/3,583	1 (Ref)	0.68 (0.51–0.90)	
By blood pressure control status^*f*^				
Controlled	555/1,233	1 (Ref)	0.81 (0.54–1.23)	0.32
Uncontrolled	185/546	1 (Ref)	0.80 (0.40–1.60)	

*CI* confidence intervals, *IDH* Isolated diastolic hypertension, *ISH* Isolated systolic hypertension, *NAFLD* non-alcoholic fatty liver disease, *OR* odds ratio, *SDH* systolic and diastolic hypertension, *VMS* vitamins and minerals supplements.

^a^All results presented in this table are sampling-weighted estimates except the number of study participants.

^b^Multivariable model was adjusted for age (quartiles), sex (male, female), household income level (quartiles), education level (elementary school, middle school, high school, college or higher), smoking status (never, past, current), alcohol drinking status (never, past, ≤ 1 time/month, 2– ≤ 4 times/month, ≥ 2 times/week), regular exercise (no, yes), body mass index (< 18.5 kg/m^2^, 18.5– < 23 kg/m^2^, 23– < 25 kg/m^2^, ≥ 25 kg/m^2^), waist circumference (cm, continuous), diabetes mellitus (no, yes), hypercholesterolemia (no, yes), number of comorbidities (0, 1, 2, 3, ≥ 4), serum triglyceride concentration (mg/dL, continuous), hypertension stage (stage1, ≥ stage2, other), current use of hypertension medications (no, yes), and intakes of total energy (quartiles) and fruits and vegetables (quartiles).

^c^P interaction was tested by including the product term between current use of VMS and each of stratification factors.

^d^Hypertension stage 1 was defined as 130 mmHg ≤ SBP < 140 mmHg or 80 mmHg ≤ DBP < 90 mmHg; and hypertension stage ≥ 2 was defined as 140 mmHg ≤ SBP or 90 mmHg ≤ DBP.

^e^ISH was defined as 130 mmHg ≤ SBP and DBP < 80 mmHg; IDH was defined as SBP < 130 mmHg and 80 mmHg ≤ DBP; SDH was defined as 130 mmHg ≤ SBP and 80 mmHg ≤ DBP.

^f^Controlled group were defined as those taking hypertension medication and having normal blood pressure levels (SBP < 130 mmHg or DBP < 80 mmHg; and uncontrolled group were defined those with hypertensive blood pressure levels despite the use of hypertension medication.

## Discussion

In this large nationally representative sample of hypertensive adults in Korea, nearly 20% reported using VMS, and current use of VMS was significantly inversely associated with odds of NAFLD. When the association of VMS use with frequency, duration, and use of multiple products was explored, the inverse association was attenuated and non-significant among those who took VMS at high frequency, for long duration, or taking multiple products. The inverse association of current VMS use did not significantly vary across most of demographics, lifestyles, and clinical characteristics, but was observed evidently only in the younger population aged <56 years or in men, but not their respective counterparts.

In our study, a majority (76.7%) of VMS users were consuming multivitamins and minerals, followed by single vitamin users (20.3%). The most commonly consumed single vitamin supplement was vitamin C (18.7%). Several evidence supports our significant inverse association between current use of VMS and NAFLD risk. In a prospective cohort of 52,280 adults in Korea, higher consumption of fruits and vegetables, major food sources of many vitamins and minerals, was significantly associated with 24% lower risk of NAFLD^38^. A recent study of 2,294 US adults in the National Health and Nutrition Examination Survey that integrated serum levels of multiple vitamins (vitamins A, B_6_, B_9_, B_12_, C, D, and E) into one index also found a significantly 41% lower NAFLD risk with increasing level of the index^13^. Similarly, the majority of previous studies of serum level^12–17,19,21–24,39–43^ or intakes^44,45^ of single nutrients in large national representative^12–17,19,21,22,24,39,42^ or other^23,40,41,43–45^ cross sectional studies showed significant inverse associations of NAFLD risk with vitamins A^45^, C^13,14,44^, B_6_^13^, B_9_^13^, D^13,15,17,21,39–41^, and E^45^, though some reported no associations with vitamin D^16,42,43^ or positive associations with vitamins A^12,13^, E^12,13^, and B_9_^19,21^, iron^23,24^, and selenium^21,22^.

At present, the underlying mechanisms for the development of NAFLD among hypertensive adults remain unclear. However, defective glucose or lipid metabolisms and/or alterations in gut microbial functions has been thought to underlie lipotoxicity of adipose tissue, inflammation, and fibrosis in the liver, which tend to progress to NAFLD^5,6^. As such, NAFLD is thought to have multifactorial metabolic pathways, including insulin resistance, oxidative stress, lipid peroxidation, and secretion of adipokines and pro-inflammatory cytokines, which are prevalent clinical conditions among hypertensive individuals^2,3^. Furthermore, abnormal activation of renin- angiotensin-aldosterone system (RAAS) that specifically occurs with hypertension may further predispose hypertensive adults to the development of NAFLD^2^. For example, hypertensive individuals tend to produce greater angiotensin from renin in the liver, which may alter hepatic lipid metabolism^2,46^. The RAAS is also found to be involved in apoptosis, reactive oxygen species production, tissue inflammation, fibrosis, and insulin resistance^46^.

The suggested hepatoprotective potential of VMS use against NAFLD risk, observed in our study, can be further explained by the involvement of vitamins and minerals in VMS in the pathogenesis of NAFLD^11^. For example, anti-oxidant vitamins and minerals, such as vitamins A, C, and E, and selenium, may reduce fibrosis in the liver by scavenging free radicals associated with lipid peroxidation, protein damage, and the induction of inflammatory cytokines such as TGF-beta, IL-6 and TNF-alpha. Vitamin D were found to reduce the secretion of fibrogenic growth factors, decrease proinflammatory cytokines, and exert an insulin-sensitizing effect favoring glucose uptake in the liver or release of adiponectin. Vitamin B3 acts as a precursor in lipid metabolisms. Folate may lower NAFLD risk via lowering homocysteine concentrations, which promote lipid accumulation. Vitamins B_2_, B_6_, B_9_ and B_12_ participate in one-carbon metabolisms that helps maintain DNA integrity and epigenetic signature. Deficiency of zinc and copper is found to augment oxidative stress. NAFLD is also considered as a hepatic component of metabolic syndrome having obesity and diabetes as strong risk factors^4^. VMS might reduce NAFLD risk by altering these metabolic conditions.

However, when we further explored associations with frequencies, durations, and multiple use of VMS to understand the optimal use of VMS, the inverse association observed with VMS use was, interestingly, attenuated and became no longer significant at high frequency (≥ 2 times/day) or long-term uses (> 16 months). Similarly, there was a significant inverse association with a single use of VMS product, but the association became null when ≥ 2 VMS products were taken. The explanation for these findings remains unclear. However, along with the widespread use of VMS, the possible risk of adverse events due to overdose and interactions with prescribed medications raises the safety concerns especially among those using VMS prevalently^47^ or with clinical disease conditions^48^.

Our results may caution the overuses of VMS against NAFLD risk among hypertensive adults. Indeed, although no studies have examined specific usages of VMS with NAFLD risk, prior studies of metabolic diseases, which tend to coexist with NAFLD^6^, have shown the harmful effects of excessive use of VMS. For instance, a previous randomized controlled trial (RCT) of 31,210 Swedish men revealed a 21–59% increased risk of age-related cataract with high doses of vitamin C (1000 mg) and E (100 mg) supplementation, which was not seen with a multivitamin supplementation containing low doses of vitamins and minerals^49^. In that study, the positive association with vitamin C supplementation was also even stronger among long-term users^49^. Similarly, a RCT of selenium supplementation (200 $\mathrm{\mu g}$/d) showed increased risk of diabetes among those in the highest tertile (> 121.6 ng/ml), but not in the lower tertiles, of plasma selenium concentration (P-interaction = 0.038)^50^. This result was further confirmed in subsequent studies^51,52^. A large cross-sectional study of 200,000 US military service members reported 2.3 to 17.9 fold risk of adverse events among those consuming ≥ 5 dietary supplements than those consuming 1–2 dietary supplements^47^. The US Preventive Services Task Force also concluded insufficient evidence to use ≥ 2 VMS products for the prevention of cardiovascular disease^53^. However, sample size in the highest categories of specific use of VMS in our study were small; individuals tended to be older and use more hypertensive medications. Our results could be due to chance or unmeasured vulnerabilities of those groups.

The inverse association between VMS use and NAFLD was only observed in adults aged < 56 year. Advancing aging is associated with diminishing nutrient absorption and progressive loss of physiological integrity. In particular, the aging was found to be associated with annual decline of hepatic clearance by 0.80% from 40 years of age, which suggests the regenerative capacity of the liver to metabolize VMS^54^. Our lack of association observed in older population might be due to loss of liver function^55^ and dysregulation of lipid and glucose metabolisms with aging, which may render body becoming less responsive to the anti-oxidants, anti-inflammatory and insulin sensitizing activities of VMS. Although no previous study on VMS use and NAFLD has examined an interaction with age, our results are consistent with those observed in the UK Biobank cohort study for cardiovascular disease outcomes, which share common risk factors and pathogenesis with NAFLD^6^. In that study of 465,268 adults, multivitamin use was inversely associated with cardiovascular disease outcomes, only among adults aged < 60 years^56^.

Furthermore, in our study VMS use was associated with a significantly lower odds of NAFLD in men, but not in women. Though the underlying mechanisms for this observation are not clear, sex-specific expression of hepatic drug metabolizing enzymes has been found^57^. Women also generally have a slower gastric emptying time and lower blood volume, liver size, and hepatic clearance than men, which might affect the absorption, distribution, bioavailability, and excretion of VMS^57,58^. Our results might suggest greater metabolizing capacity of VMS among men than women, aligning with the well-known sex difference in pharmacokinetics^58,59^. In addition, men have greater hepatic insulin insensitivity and visceral adiposity than women^60^. These sex-specific biological variations associated with the pathogenesis of NAFLD might also predispose men to greater metabolic stress and lipoapoptosis in hepatocytes^60^, resulting in a greater benefit from VMS for men than women.

Our study has several strengths. To the best of our knowledge, ours is the first study that comprehensively evaluated the association between VMS use and NAFLD among hypertensive adults, and thus has highly significant clinical implication. We had detailed data on VMS usages and were able to explore associations, beyond the current status of VMS, including frequencies, duration, and use of multiple products. We comprehensively adjusted for potential confounding factors for NAFLD and conducted stratified analyses by demographics, lifestyle, and clinical conditions.

However, our cross-sectional study design limits to infer causality. Nonetheless, NAFLD is asymptomatic. Individuals are less likely to change VMS use recognizing NAFLD status, though we cannot confirm this due to lack of data on change of VMS use. Second, NAFLD status was not ascertained by ultrasound or biopsy, the gold standard for diagnosing NAFLD. However, HSI has high sensitivity and specificity^32^, and is considered the most appropriate index for Asian populations^32^. Third, the distribution of VMS frequency, duration, and use of multiple VMS in our study was skewed limiting our power on examining appropriate use of VMS. Fourth, we did not have details on prescribed drugs, which may have an interaction with VMS, although we observed no significant effect modification by hypertension stage or hypertension medication use. Fifth, our study population are hypertensive adults, limiting generalizability of our findings. Finally, we cannot rule out unmeasured or residual confounding.

In conclusion, in this first large nationally representative study of hypertensive adults, current use of VMS was significantly associated with lower risk of NAFLD. The inverse association was stronger in younger than older individuals and in men than women. The attenuation of inverse association with current use of VMS at higher frequency, with longer duration of use, and with use of multiple products corroborates the positional statements of health authorities, which cautions overuses of VMS despite its potential benefit^48,53^. Large-scale cohort studies are warranted to replicate our finding.

### Supplementary Information

Supplementary Information.

## Data Availability

Regarding to the datasets used and/or analyzed during the current study, the datasets used and/or analyzed will be available from the corresponding author on reasonable request.

## Abbreviations

ALT: Alanine aminotransferase
AST: Aspartate aminotransferase
BMI: Body mass index
CI: Confidence intervals
HDL-C: High density lipoprotein cholesterol
HIS: Hepatic steatosis index
KCDC: The Korea centers for disease control and prevention
KNHANES: The Korea national health and nutrition examination survey
M_VMS: Multivitamin or multimineral supplements
MVOR: Multivariable-adjusted odds ratio
NAFLD: Non-alcoholic fatty liver disease
OR: Odds ratio
RAAS: Renin–angiotensin–aldosterone system
S_MIN: Single-mineral supplements
S_VIT: Single-vitamin supplements
VMS: Vitamin and mineral supplements
