# Supplementary material for: Association between use of vitamin and mineral supplement and non-alcoholic fatty liver disease in hypertensive adults
Source: Sci Rep. 2023 Aug 22;13:13670. doi: 10.1038/s41598-023-40868-1 (PMC10444877; doi:10.1038/s41598-023-40868-1)
Supplement: Supplementary file 1 — Supplementary Information. [file 41598_2023_40868_MOESM1_ESM.pdf]

Title: Association between use of vitamin and mineral supplement and non-alcoholic fatty liver disease in hypertensive adults

Yoonmi Park, MS[1,2], Stephanie A. Smith-Warner, PhD[3,4], Xuehong Zhang, ScD[3,5], Yoon Jung Park, PhD[1,2], Hyesook Kim, PhD[6], Hyesook Park, MD, PhD[2,7], Hye Ah Lee, PhD[8], Seungyoun Jung, ScD[1,2*]

[1]Department of Nutritional Science and Food management, Ewha Womans University, Seoul, Republic of Korea

[2]Graduate Program in System Health Science and Engineering, Ewha Womans University, Seoul, Republic of Korea

[3]Department of Nutrition, Harvard T. H. Chan School of Public Health, Boston, MA, USA.

[4]Department of Epidemiology, Harvard T. H. Chan School of Public Health, Boston, MA, USA

[5]Channing Division of Network Medicine, Department of Medicine, Brigham and Women's Hospital and Harvard Medical School, Boston, MA, USA.

[6]Department of Food and Nutrition, Wonkwang University, Jeonbuk Republic of Korea

[7]Department of Preventive Medicine, College of Medicine, Ewha Womans University, Seoul k, Republic of Korea

[8]Clinical Trial Center, Mokdong Hospital, Ewha Womans University, Seoul, Republic of Korea

Corresponding author

Dr. Seungyoun Jung

Department of Nutritional Science and Food management, Graduate Program in System Health Science and Engineering Ewha Womans University, Seoul, Korea 52, Ewha womans university, Seodaemun-gu, Seoul, 03760

Tel: +82-02-2627

Email: sjung131@ewha.ac.kr

Table S1. Types of vitamin and mineral supplements (VMS) used among current VMS users (N=1,373)

VMS type	%
**Multivitamin or multimineral (M_VMS)[a]**	**76.67**
**Single vitamin (S_VIT)[b]**	**20.32**
Vitamin A	0.19
Vitamin B_1	0.09
Vitamin B_3	0.09
Vitamin C	18.67
Vitamin D	0.19
Vitamin E	1.09
**Single mineral (S_MIN)[c]**	**3.01**
Calcium	1.49
Iron	0.74
Selenium	0.29
Zinc	0.49

[a] M_VMS use was defined as taking products containing two or more vitamins and/or minerals; of these, 22.2% were using ≥2 different VMS products.

[b] S_VIT use was defined as taking a supplement containing one vitamin

[c] S_MIN use was defined as taking a supplement containing one mineral

Table S2. Multivariable-adjusted[a,b] odds ratio (OR) and 95% confidence intervals (95% CIs) of NAFLD according to type of vitamin and mineral supplements (VMS)

VMS type	Current use of VMS	
	Nonusers OR (95%Cl)	Current users OR (95%Cl)
**By VMS type**		
Multivitamin or multimineral supplement (M_VMS)[c]	1 (Ref)	0.76 (0. 58-0.99)
Single vitamin supplement (S_VIT)[d]	1 (Ref)	0.72 (0.46-1.13)
Single mineral supplement (S_MIN)[e]	1 (Ref)	0.34 (0.12-0.93)

Abbreviations: CI, confidence intervals; M_VMS, multivitamin or multimineral supplement; NAFLD, non-alcoholic fatty liver disease; OR, odds ratio; S_MIN, single mineral supplement; S_VIT, single vitamin supplement; VMS, vitamin and mineral supplements

[a]All results presented in this table are sampling-weighted estimates except the number of study participants

[b] Multivariable model was adjusted for age (quartiles), sex (male, female), household income level (quartiles), education level (elementary school, middle school, high school, college or higher), smoking status (never, past, current), alcohol drinking status (never, past, ≤1 time/month, 2-≤4 times /month, ≥ 2 times/week), regular exercise (no, yes), body mass index (< 18.5kg/m^2, 18.5- < 23kg/m^2, 23- < 25kg/m^2, ≥25kg/m^2), waist circumference (cm, continuous), diabetes mellitus (no, yes, missing), hypercholesterolemia (no, yes), number of comorbidities (0, 1, 2, 3, ≥4), serum triglyceride concentration (mg/dL, continuous), hypertension stage (stage1, ≥stage2, other), current use of hypertension medications (no, yes), and intakes of total energy (quartiles) and fruits and vegetables (quartiles)

[c] This analysis was conducted among M_VMS users (n= 1,073) and nonusers (n=6,003), of which 1,949 were NAFLD cases.

[d] This analysis was conducted among S_VIT users (n=257) and nonusers (n=6,003), of which 1,775 were NAFLD cases.

[e] This analysis was conducted among S_MIN users (n=43) and nonusers (n=6,003), of which 1,719 were NAFLD cases.

Table S3. Multivariable-adjusted[a,b] odds ratio (OR) and 95% confidence intervals (95% CIs) of NAFLD according to use of vitamin and mineral supplement (VMS) according to macronutrient consumption and eating habit

Stratification factors	Cases/ Non-cases	Use of VMS		P Interaction[c]
		Nonusers OR (95% CI)	Current users OR (95% CI)	
**By percentage of fat[d,f]**				
Fat < 13.5	915/2,773	1 (Ref)	1.02 (0.70-1.50)	0.13
Fat ≥ 13.5	1,100/2,588	1 (Ref)	0.58 (0.43-0.78)	
**By percentage of carbohydrate[d,f]**				
Carbohydrate < 71.0	1,043/2,645	1 (Ref)	0.66 (0.49-0.89)	0.41
Carbohydrate ≥ 71.0	972/2,716	1 (Ref)	0.84 (0.59-1.19)	
**By percentage of protein[d,f]**				
Protein < 13.5	939/2,749	1 (Ref)	0.91 (0.66-1.26)	0.32
Protein ≥ 13.5	1,076/2,612	1 (Ref)	0.61 (0.44-0.84)	
**By eating frequency[e]**				
Eating frequency < 3	632/1,296	1 (Ref)	0.27 (0.39-0.99)	0.99
Eating frequency = 3	1,383/4,065	1 (Ref)	0.79 (0.61-1.03)	

Abbreviations: BMI, body mass index; CI, confidence intervals; NAFLD, non-alcoholic fatty liver disease; OR, odds ratio; VMS, vitamin and mineral supplements

[a] All results presented in this table are sampling-weighted estimates except the number of study participants

[b] Multivariable model was adjusted for age (quartiles), sex (male, female), household income level (quartiles), education level (elementary school, middle school, high school, college or higher), smoking status (never, past, current), alcohol drinking status (never, past, ≤1 time/month, 2-≤4 times/month, ≥2 times/week), regular exercise (no, yes), body mass index (< 18.5kg/m^2, 18.5-< 23kg/m^2, ≥23-< 25kg/m^2, ≥25kg/m^2), waist circumference (cm, continuous), diabetes mellitus (no, yes), hypercholesterolemia (no, yes), number of comorbidities (0, 1, 2, 3, ≥4), serum triglyceride concentration (mg/dL, continuous), hypertension stage (stage1, ≥stage2, other), current use of hypertension medications (no, yes), and intakes of total energy (quartiles) and fruits and vegetables (quartiles)

[c] P interaction was tested by including the product term between current use of VMS and each of stratification factors.

[d] Percentage of fat, carbohydrate, and protein were categorized using the median value of our study population as a cut-off value

[e] Eating frequency is the average of the meal frequencies reported for one day prior to the survey and two days prior to the survey

[f] Fat, carbohydrate, and protein intake were categorized using the median values of our study participants, while meal frequency was derived by averaging the reported number of meals over the preceding two days.
